# Evaluation of the Alpha-Fetoprotein Model for Predicting Recurrence and Survival in Patients With Hepatitis B Virus (HBV)–Related Cirrhosis Who Received Liver Transplantation for Hepatocellular Carcinoma

**DOI:** 10.3389/fsurg.2020.00052

**Published:** 2020-08-21

**Authors:** Ao Ren, Zhongqiu Li, Xiaozhuan Zhou, Xuzhi Zhang, Xiaochun Huang, Ronghai Deng, Yi Ma

**Affiliations:** ^1^Organ Transplant Center, The First Affiliated Hospital, Sun Yat-sen University, Guangzhou, China; ^2^Guangdong Provincial Key Laboratory of Organ Donation and Transplant Immunology, The First Affiliated Hospital, Sun Yat-sen University, Guangzhou, China; ^3^Guangdong Provincial International Cooperation Base of Science and Technology (Organ Transplantation), The First Affiliated Hospital, Sun Yat-sen University, Guangzhou, China

**Keywords:** alpha-fetoprotein (AFP) model, liver transplantation, hepatocellular carcinoma, hepatitis B virus, liver cirrhosis

## Abstract

**Introduction:** The alpha-fetoprotein (AFP) model is superior to the Milan criteria in predicting the recurrence of hepatocellular carcinoma (HCC) after liver transplantation in European and Latin American populations. The purpose of this study was to determine the predictive value of the AFP model in Chinese hepatitis B virus (HBV)–related cirrhosis HCC patients.

**Methods:** A total of 189 patients with HBV-related cirrhotic HCC were included. The recurrence rate and survival rate were estimated, and predictability was assessed by the Net Reclassification Improvement (NRI) method.

**Results:** Of the 189 patients, patients with an AFP score >2 had a higher recurrence rate at 5 years (48.94 vs. 13.53%, *p* < 0.05) and lower survival rate (43.96 vs. 68.97%, *p* < 0.05). Considering patients within the Milan criteria, a higher 5-year recurrence rate and lower survival rate were observed in patients with an AFP model score >2 points compared to patients with a score of ≤ 2 points (recurrence rate: 58.75 vs. 12.98%, *p* < 0.05; survival rate: 28.57 vs. 67.41%, *p* = 0.047). NRI analysis showed that the AFP model exhibited superior predictability as compared to the Milan criteria.

**Conclusions:** The AFP model may be used as a selection tool for Chinese HBV patients who require liver transplantation due to HCC.

## Introduction

Hepatocellular carcinoma (HCC) is the fifth most common cancer and third most common cause of cancer-related mortality (1). A major cause is chronic hepatitis B virus (HBV) infection, especially in China (2). Liver transplantation (LT) is considered an effective treatment for liver cirrhosis and HCC. However, the efficacy is limited by the risk of HCC recurrence, which negatively affects patient survival. The HCC recurrence rate is reported to range from 30 to 40% (3, 4). Since the Milan criteria were established, the HCC recurrence rate after LT has been steadily decreasing (5, 6). Since the Milan criteria were established, other criteria have been proposed (7–9), such as the University of California San Francisco (UCSF) criteria, the Hangzhou criteria, and the Up-to-Seven criteria. With the expansion of criteria, the percentage of patients with HCC eligible for LT increased from ~16 to 51% (10). However, HCC recurrence is still the main cause of mortality after LT; thus, existing criteria require further improvement (11).

The Milan criteria and other criteria mainly consider tumor burden, metastasis, and vascular invasion. Recently, more focus has been placed toward using other clinical factors as selection tools. One is alpha-fetoprotein (AFP) level, as an elevated AFP level before LT has been associated with higher recurrence rates (9, 12, 13). In 2012, a French study group proposed new selection criteria for HCC transplant candidates called the AFP model, and it has been shown to be superior to the Milan criteria in French, Italian, and Latin America populations (14–17). The AFP model is based on tumor stage and AFP values, and assigns values to the diameter and number of nodules and AFP value. The model score varies between 0 and 9 points, and values below the cutoff of 2 points identify patients with a high survival rate and low recurrence rate (14).

In China, cirrhosis caused by HBV is the primary cause of HCC; however, alcoholic cirrhosis and hepatitis C virus (HCV) are the most important causes of HCC in the French population (14). In the Latin America population, the AFP model performed better in non-HBV patients, and in the Italian population, the AFP model performed better in HCV patients (15, 16). As such, new prediction models need to be evaluated in different populations with different distributions of underlying liver diseases.

To the best of our knowledge, no study has examined the predictive value of the AFP model in an Asian population. The aim of this study was to determine the predictive value of the AFP model for recurrence and survival in a Chinese HBV-related cirrhosis HCC population, and compare the results to the Milan criteria.

## Patients and Methods

The records of 189 patients with HBV-related cirrhosis who received an LT for HCC at the First Affiliated Hospital of Sun Yat-sen University (Guangzhou, China) from 2010 to 2015 were retrospectively reviewed. Inclusion criteria were as follows: (1) histopathologic proof of HCC on the explanted liver and (2) venous or extrahepatic tumor involvement not found on preoperative ultrasound or computed tomography (CT) scan examination. Exclusion criteria were as follows: (1) incidental HCC, (2) patients younger than 18 years of age, (3) venous or extrahepatic tumor involvement found on pre-LT images, and (4) retransplantation. The diagnosis was confirmed by medical imaging, seropositivity for hepatitis B surface antigen (HBsAg), and pathological examination of tissue specimens. Immunosuppressive therapy for all patients after LT was individualized therapy and was mainly based on Simulect and tacrolimus, combined with other immunosuppressive agents. Drug dosages were adjusted based on drug blood concentrations.

Recipient data extracted from the medical records included age and sex, model of end-stage liver disease (MELD) score, Child–Pugh classification, HBV infection status, preoperative AFP level, imaging features, and pretransplant treatments. Mortality and recurrence data were also extracted, as well as follow-up time.

The AFP score was calculated for patients at the last evaluation before LT, using the simplified version of the AFP model (Table 1) (14). Tumor characteristics were based on pre-LT magnetic resonance image (MRI) or CT. HCC pathological features were based on pathology reports after LT. Patients were followed until death, or until they were lost to follow-up. Follow-up studies included CT, MRI, bone scintigraphy, and serum AFP level. Recurrence was determined by imaging results plus AFP level or tissue biopsy.

**Table 1:** Simplified version of the alpha-fetoprotein (AFP) model.

**Variables**	**β coefficient**	**Hazard ratio (HR)**	**Points**
Largest diameter			
≤ 3 cm	0	1	0
3–6 cm	0.272	1.31	1
> 6 cm	1.347	3.84	4
Number of nodules			
1–3	0	1	0
4 or more	0.696	2.01	2
AFP level (ng/ml)			
≤ 100	0	1	0
100–1,000	0.668	1.95	2
>1,000	0.945	2.57	3

*In Duvoux et al. (14)*.

*In this simplified version, the score was calculated by adding the individual points for each obtained variable. A cutoff value of 2 separated patients at high and low risk of recurrence. The cutoff value of 2 selected exactly the same patients as the original Cox score of 0.7 as a cutoff value*.

Fisher's exact test or chi-square test was used for comparisons of categorical data. Student's *t-*test and the Mann–Whitney *U-*test were used for the comparisons of continuous variables, according to their distribution, as appropriate. Survival curves were generated by the Kaplan–Meier method, and hazard ratios (HRs) for HCC recurrence were calculated using Cox regression multivariate analysis. Net Reclassification Improvement (NRI) and recurrence rates for patients within and beyond the Milan criteria were estimated according to the new model (16). All statistical analyses were performed using SPSS version 19.0 statistical software (SPSS, Chicago, IL, USA). Values of *p* < 0.05 were considered to indicate statistical significance.

All organs come from voluntary donation from citizens; no executed prisoner was involved. The study was approved by the Institutional Review Board of the First Affiliated Hospital of Sun Yat-sen University and in accordance with the Declaration of Istanbul. All protocols conformed to the ethical guidelines of the 1975 Helsinki Declaration.

## Results

During the study period, 530 adults received an LT; 295 of these patients had HCC. Of the 295 patients, 189 had HBV-related cirrhosis and were included in the study. Patient demographic and tumor characteristics are summarized in Table 2.

**Table 2:** Baseline characteristics of the patients.

**Variable**	**Values**
Age (years) (median [interquartile range, IQR])	52 [45–59]
Gender, male, n (%)	175 (92.59)
Model of end-stage liver disease (MELD) score (median [IQR])	10 (7-16)
Child–Pugh A/B/C, n (%)	25 (13.23)/127 (67.20)/37 (19.58)
Tumors within Milan criteria, n (%)	81 (42.86)
AFP level (ng/ml), n (%)	
AFP ≤100 ng/ml, n (%)	98 (51.85)
AFP 100–1,000 ng/ml, n (%)	39 (20.64)
AFP >1,000 ng/ml, n (%)	52 (27.51)
Number of nodules (median [IQR])	1 (1-4)
Maximal diameter of the largest tumor (cm) (median [IQR])	3.9 [2.5–6.2]
Bridging therapies, n (%)	102 (53.97)
AFP score: ≤2 vs. >2, n (%)	88 (46.56) vs. 101 (53.44)
Follow-up (months) (median [IQR])	43 (13-64)

Of the 189 patients, 92.6% were male, and the median age of the 189 patients was 52 years [interquartile range (IQR): 45–59 years]. Overall, 42.86% (*n* = 81) of the patients were within the Milan criteria. The median MELD score was 10 (IQR: 7–16). Of the patients, 127 (67.2%) were Child–Pugh class B. Ninety-eight (51.85%) patients had an AFP level ≤ 100 ng/ml. The median maximal tumor diameter was 3.9 cm (IQR: 2.5–6.2 cm), and the median number of nodules was 1 (IQR: 1–4). One hundred and two (53.97%) patients received bridging therapy prior to transplantation: transarterial chemoembolization (TACE) in 73 patients, radiofrequency ablation (RFA) in 20 patients, and surgical resection in 16 patients. The median follow-up time was 43 months (IQR: 13–64 months).

Based on the AFP model, clinicopathological characteristics were compared between patients whose AFP model scores were ≤2 or >2 (Table 3): 101 patients (53.4%) had an AFP model score >2. The two groups were similar with respect to age, gender, MELD score, Child–Pugh class, and pretransplant treatment. All patients received antiviral therapy before and after LT.

**Table 3:** Patients' characteristics at listing in the two cohorts according to the cutoff value 2 of AFP model.

**Variable**	**AFP score ≤ 2 (88)**	**AFP score >2 (101)**	***P***
Age (years) (median [IQR])	53 [47–60]	50 [43–59]	*p =* 0.112
Gender, male, n (%)	80 (90.91)	95 (94.06)	*p =* 0.579
MELD score (median, [IQR])	10 (7-16)	10 (7-16)	*p =* 0.83
Child–Pugh A/B/C, n (%)	11 (12.5)/63 (71.59)/14 (15.91)	14 (13.86)/64 (63.37)/23 (22.77)	*p =* 0.434
Tumors within Milan criteria, n (%)	69 (78.41)	12 (11.88)	*p =* 0.000
Bridging therapies, n (%)	49 (55.68)	53 (52.48)	*p =* 0.664
Micro-vascular invasion, n (%)	12 (13.64)	45 (44.55)	*P* = 0.000
Macro-vascular invasion, n (%)	2 (2.27)	9 (8.91)	*P* = 0.048
Poorly differentiated tumor, n (%)	12 (13.64)	25 (24.75)	*P* = 0.018

However, there were some significant differences between groups with an AFP model score ≤ 2 and >2. The group with an AFP model score >2 had a higher proportion of patients with micro-vascular invasion and macro-vascular invasion and with pathological stages of tumor.

**Figure 1:**
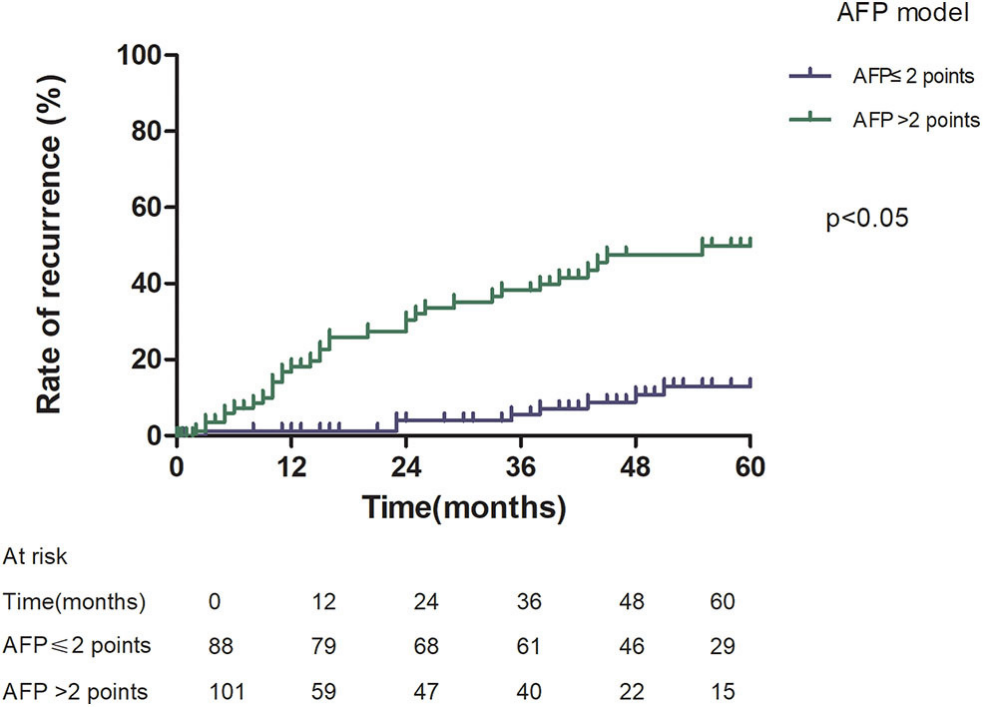
Risk of recurrence according to the alpha-fetoprotein (AFP) score cutoff of 2.

**Figure 2:**
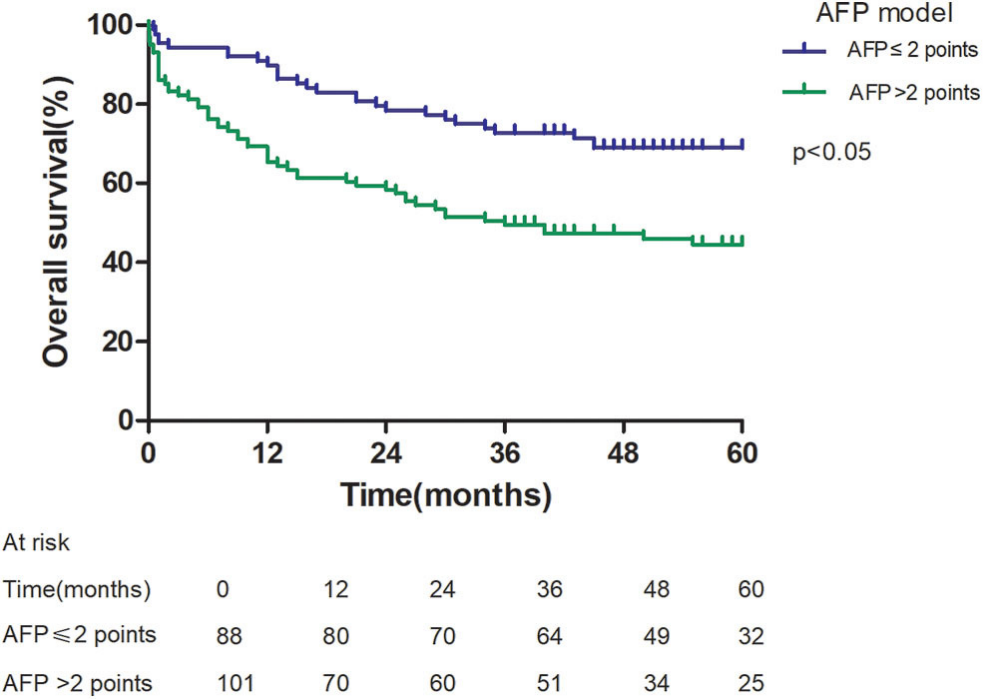
Overall survival according to the AFP score cutoff of 2.

**Figure 3:**
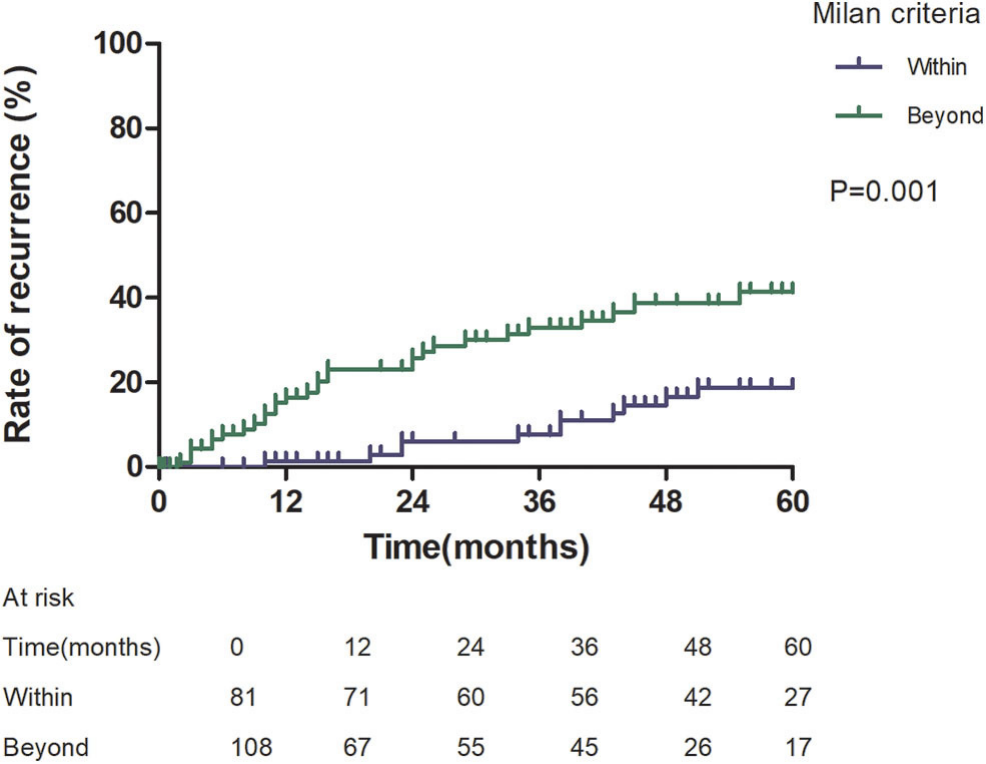
Risk of recurrence according to Milan criteria.

**Figure 4:**
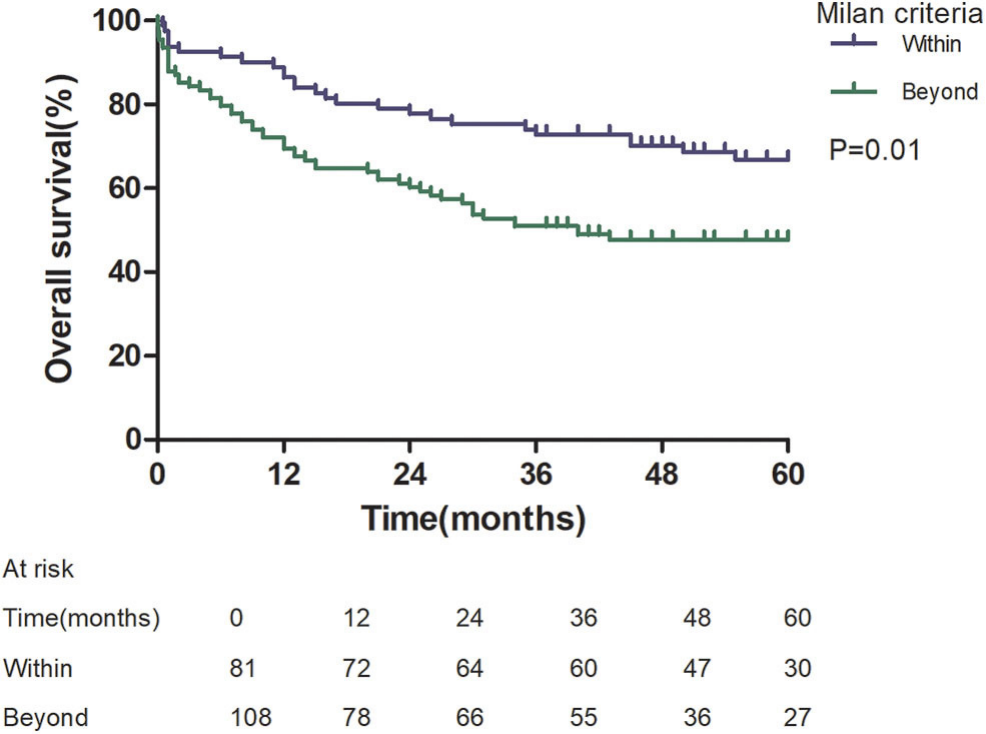
Overall survival according to Milan criteria.

Patients with an AFP model score >2 had a higher 5-year recurrence rate (48.94 vs. 13.53%, *p* < 0.05; Figure 1) and a lower 5-year survival rate (43.96 vs. 68.97%, *p* < 0.05; Figure 2) compared to those with a score of ≤2 points. The recurrence rates of patients within and beyond the Milan criteria were 19.36 and 40.46%, respectively (*p* = 0.001; Figure 3), with corresponding 5-year survival rates of 62.21 and 47.57%, respectively (*p* = 0.001; Figure 4).

**Figure 5:**
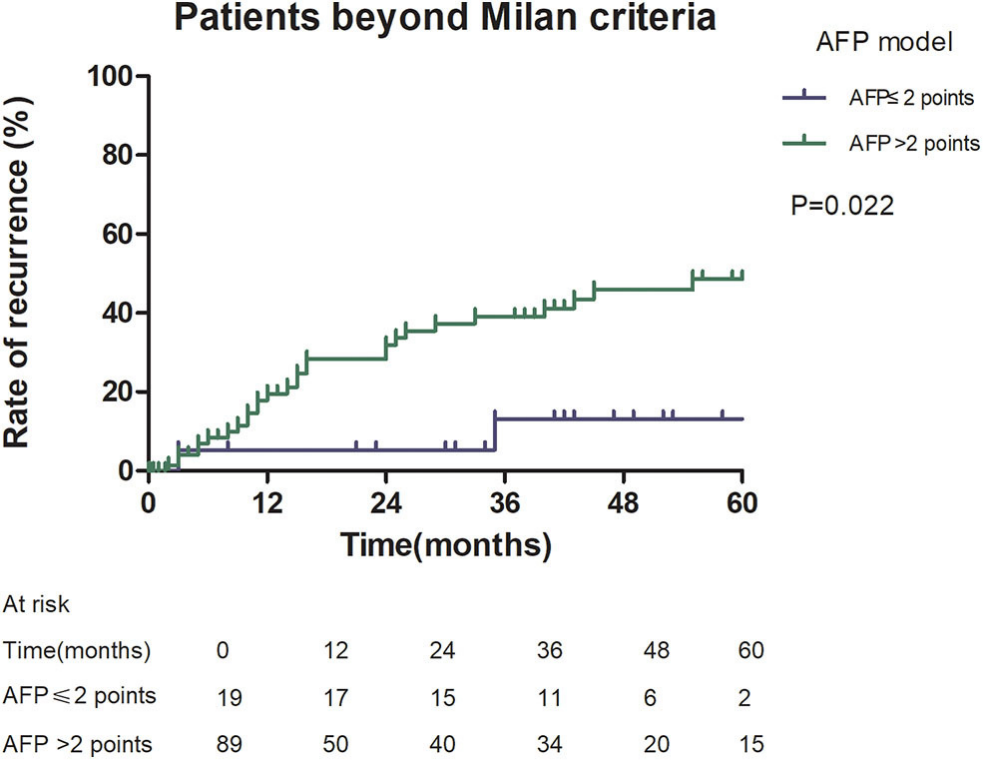
Risk of recurrence according to the AFP score cutoff of 2, in patients beyond Milan criteria.

**Figure 6:**
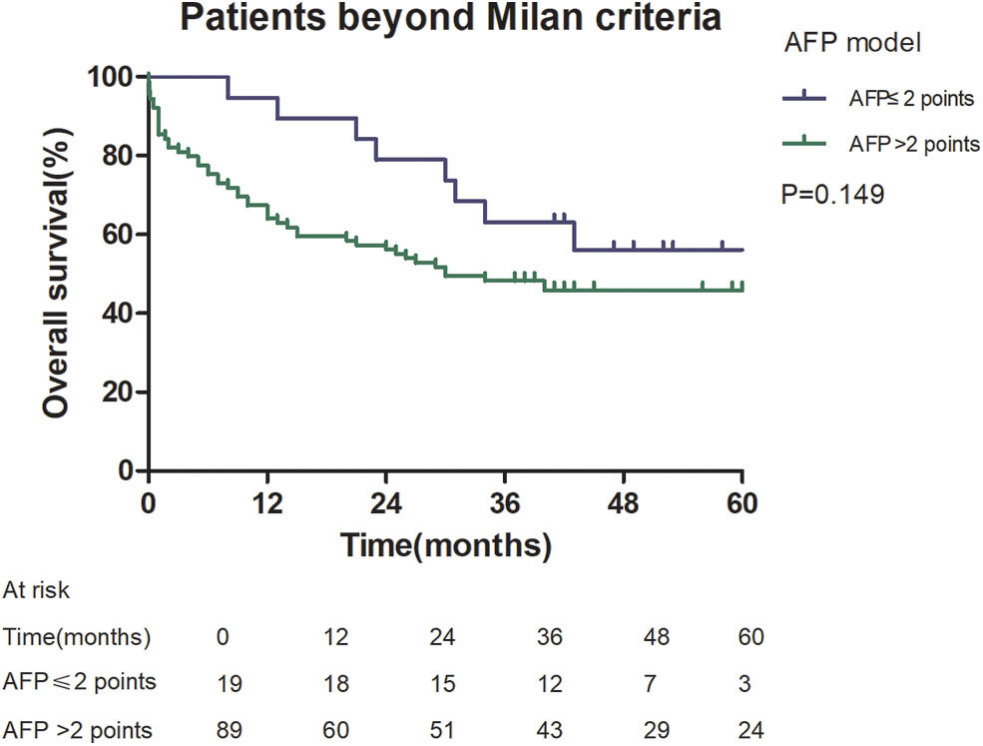
Overall survival according to the AFP score cutoff of 2, in patients beyond Milan criteria.

**Figure 7:**
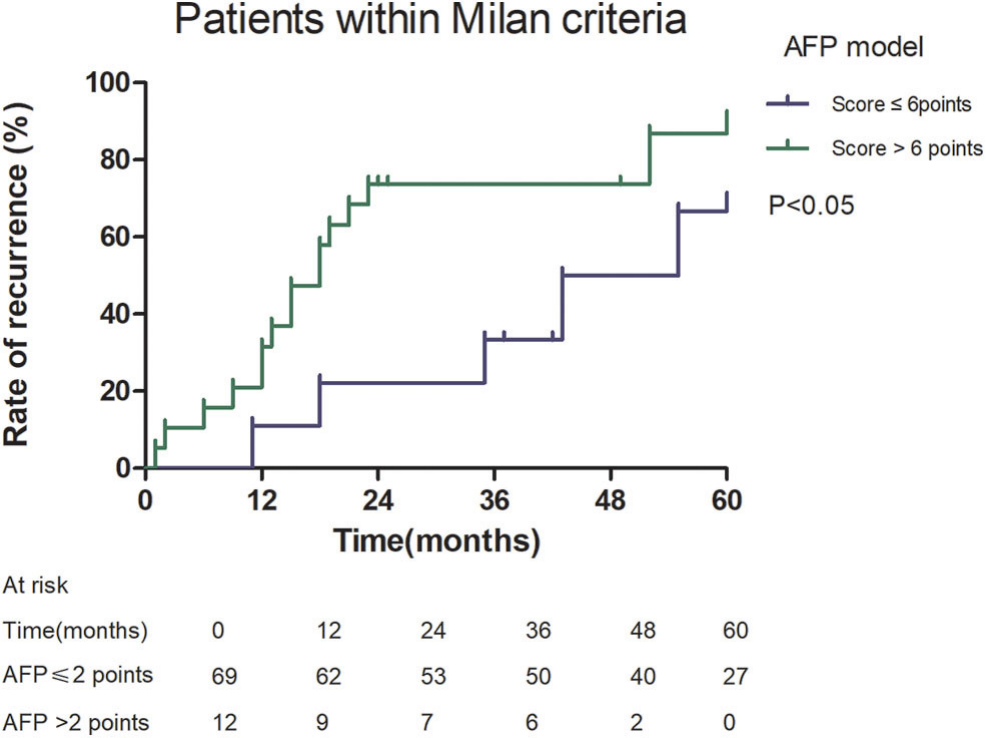
Risk of recurrence according to the AFP score cutoff of 2, in patients within Milan criteria.

**Figure 8:**
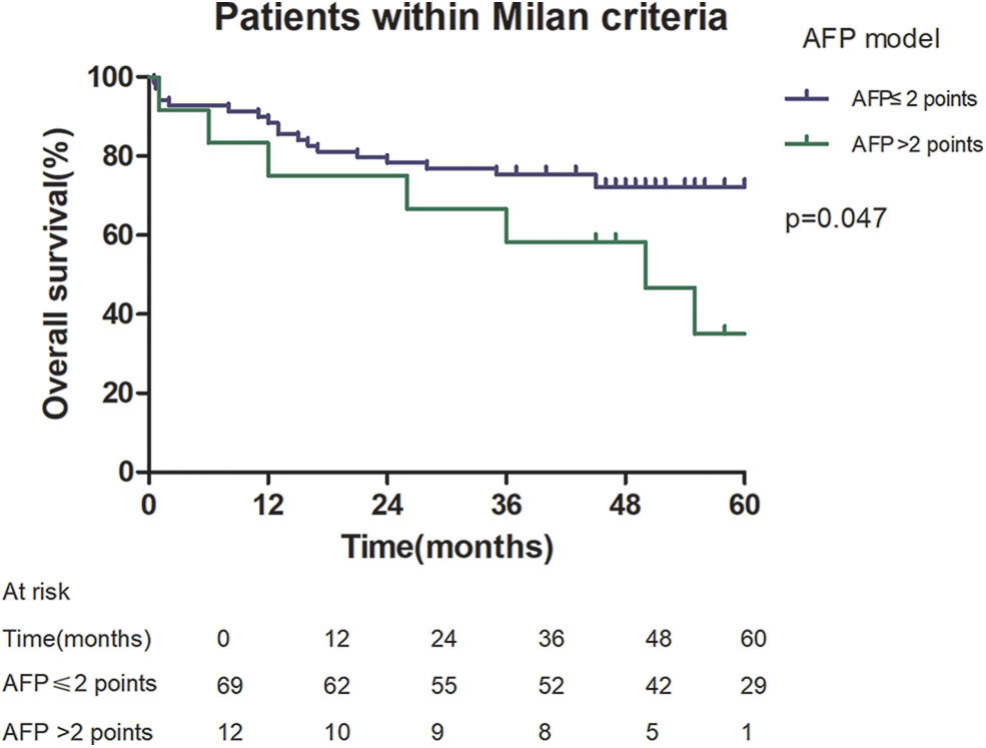
Overall survival according to the AFP score cutoff of 2, in patients within Milan criteria.

Among patients exceeding the Milan criteria, the 5-year recurrence rate was higher in patients with an AFP model score >2 points compared to those with a score of ≤ 2 points (47.14 vs. 12.42%, *p* = 0.022; Figure 5). The 5-year survival rate was higher in patients exceeding the Milan criteria with an AFP model score ≤ 2 points compared to patients with a score of > 2 points (56.84 vs. 45.71%); however, the difference was not statistically significant (*p* = 0.149; Figure 6). Considering patients within the Milan criteria, a higher 5-year recurrence rate and lower survival rate were observed in patients with an AFP model score >2 points compared to patients with a score of ≤ 2 points (recurrence rate: 58.75 vs. 12.98%, *p* < 0.05; Figure 7; survival rate: 28.57 vs. 67.41%, *p* = 0.047; Figure 8).

NRI analysis indicated that the AFP model significantly improved the classification of patients without recurrence compared to the Milan criteria (NRI = 0.141, *z* = 1.94, *p* = 0.026). This indicated that the prediction of recurrence was improved significantly by the AFP model compared with the Milan criteria.

## Discussion

Since the introduction of the AFP model by Duvoux et al. (14) in 2012, better clinical outcomes after LT have been achieved for European and Latin American HCC patients compared to the Milan criteria. However, whether the AFP model is practical for other populations still needs to be explored. Our results in an Asian population showed that the AFP model discriminated better between patients with low and high risk of recurrence, resulting in a significant impact on patient survival, just as in European and Latin American populations (14–17). It is noteworthy that the HCC patients in our study differed from the European cohorts because HBV-related cirrhosis was the main cause of HCC in our patients, which is similar to Latin American populations. In French patients, alcoholic cirrhosis was the most important cause of HCC, and in Italian patients, HCV-related cirrhosis was the primary cause of HCC (14–17). In Latin American patients, the AFP model performed better for predicting HCC recurrence in non-HBV patients (*p* < 0.0001 vs. *p* = 0.15); however, the 5-year risk of recurrence was statistically significant according to AFP score in Italian patients with HBV. HBV-related HCC is a considerable worldwide problem; HBV infection accounts for about 50% of HCC cases worldwide, and in East Asia, more than 70% (1, 18, 19). It is important to verify whether the AFP model is an effective prediction tool for HBV-related cirrhotic HCC.

This study was a retrospective study, similar to previous studies. All cases were HCC complicated HBV-related cirrhosis. This was significantly different from the cause of HCC in French, Italian, and Latin American cohorts (14–16). In our study, patients with an AFP model score >2 had a higher 5-year recurrence rate (48.94 vs. 13.53%, *p* < 0.05) and lower 5-year survival rate (43.96 vs. 68.97%, *p* < 0.05) compared to those with a score of ≤ 2 points. This showed that the AFP model could accurately distinguish Asian HCC patients with HBV-related cirrhosis with low recurrence risk and high recurrence risk, results similar to those seen in Italian patients. However, results in a Latin American cohort suggested that the AFP model performed better in non-HBV patients for the prediction of HCC recurrence after LT. Among patients exceeding the Milan criteria, cutoff values of > 2 points in the AFP model further identified a subgroup of patients with a higher risk of recurrence (47.14 vs. 12.42%, *p* = 0.022) and 45.71% 5-year survival. Similar results were observed in patients within the Milan criteria, with a higher risk of recurrence and lower 5-year survival among patients with an AFP model score >2. According to NRI analysis, the AFP model significantly improved the classification of patients without recurrence compared to the Milan criteria (NRI = 0.141, *z* = 1.94, *p* = 0.026), which indicated that the prediction of recurrence was improved significantly by the AFP model compared with the Milan criteria.

The AFP model combined AFP level with tumor size and number. Several studies have shown that AFP level is an independent predictor of recurrence after LT for HCC and also predicts post-LT survival (14, 20, 21). Our results also showed that patients with a high AFP level have a high risk for recurrence. The AFP model was proven to be superior to the Milan criteria in European and Latin American cohorts. It is worth noting that the AFP model identified a subgroup of patients who exceeded the Milan criteria who had good outcomes, which may be related to incorporating AFP level into the selection process (22). The group with an AFP model score > 2 had a higher proportion of patients with micro-vascular invasion and macro-vascular invasion, and poorly differentiated tumors. Numerous previous studies have showed that recurrence is associated with aggressive tumor pathological characteristics, such as large tumor size, multiple tumors, poor differentiation, and vascular invasion (23, 24). Vascular invasion and poor differentiation of HCC were closely related to poor prognosis (25). Therefore, the AFP model performed better for predicting HCC recurrence.

This was a retrospective, single-center study with a small number of patients and with a long inclusion period, which might limit the reproducibility of our result. The multi-regional multicenter design allowed obtaining a larger cohort of patients with longer follow-up periods, which might be crucial for a more precise evaluation of this model for predicting the prognosis of HBV-related cirrhotic HCC after LT. Although more accurate for predicting recurrence than MC, the AFP score is worth further improvement. Some patients with an AFP score >2 do not experience recurrence. New predictive models combined with pathological or molecular tools or detection of circulating tumor cells may better predict tumor recurrence after LT in the future.

In conclusion, the AFP model performed better than the Milan criteria for predicting recurrence and survival after LT in a Chinese population with HBV-related cirrhosis. This important finding strongly supports the adoption of the AFP model as a selection tool for HCC patients in programs with HBV-related cirrhosis, which may be useful in Asian countries.

## Data Availability Statement

The raw data supporting the conclusions of this article will be made available by the authors, without undue reservation, to any qualified researcher.

## Ethics Statement

The studies involving human participants were reviewed and approved by the Institutional Review Board of The First Affiliated Hospital of Sun Yat-sen University. The patients/participants provided their written informed consent to participate in this study. Written informed consent was obtained from the individual(s) for the publication of any potentially identifiable images or data included in this article.

## Author Contributions

AR and ZL designed the study, collected and analyzed the data, wrote, and revised the manuscript. XZho, XZha, XH, and RD participated in the study design and data acquisition. YM contributed to the conception and design of the study as well as funding application. All authors have approved the final version to be published.

## Conflict of Interest

The authors declare that the research was conducted in the absence of any commercial or financial relationships that could be construed as a potential conflict of interest.
